# Differential functions of ROCK1 and ROCK2 in liver fibrosis and advances in targeted therapeutic strategies

**DOI:** 10.3389/fphar.2026.1929302

**Published:** 2026-09-29

**Authors:** Ting He, Yinqi Li, Zhihan Chen, Jiemei Chen

**Affiliations:** 1 School of Pharmacy, Guangxi Medical University, Nanning, Guangxi, China; 2 Department of Pharmacy, The First Affiliated Hospital of Guangxi Medical University, Nanning, Guangxi, China

**Keywords:** liver fibrosis, Rho‐associated coiled‐coil containing protein kinase (ROCK), Rho/ROCK signaling pathway, selective ROCK2 inhibitor, hepatic stellate cells, liver sinusoidal endothelial cells, targeted drug delivery.

## Abstract

Liver fibrosis, driven by metabolic dysfunction-associated steatohepatitis (MASH) and other chronic injuries, represents a global health crisis without approved pharmacological therapies. The Rho-associated coiled-coil kinases (ROCK1 and ROCK2), the principal effectors of Rho GTPases, have emerged as central nodes in fibrogenesis, yet the isoform-specific mechanisms and their translational potential remain incompletely defined. This review systematically dissects the divergent roles of ROCK1 and ROCK2 across the hepatic cellular landscape. We synthesize current knowledge on isoform-specific functions across major hepatic cell types, including hepatic stellate cells (HSCs), liver sinusoidal endothelial cells (LSECs), hepatocytes, macrophages, and immune cells, with emphasis on the evidence quality underlying each claim. ROCK2 appears to play a predominant role in the profibrotic vascular niche in several contexts, particularly in LSEC capillarization and HSC contraction; however, the contributions of both isoforms are fundamentally cell- and context-dependent. We discuss the evidence for ROCK isoform-specific functions, distinguishing among genetic, pharmacological, expression-based, and activation-based evidence. We further synthesize the complex crosstalk between ROCK signaling and the TGF-β/Smad, Wnt/β-catenin, Hippo/YAP, and NOX4/ROS networks. Crucially, we critically evaluate the translational pipeline—from pan-ROCK inhibitors limited by systemic hypotension to next-generation selective ROCK2 inhibitors. A notable development in clinical translation is the selective ROCK2 inhibitor TDI01, which targets endothelial ROCK2 to attenuate fibrosis. In an extended, single-arm, open-label study of TDI01 in patients with liver fibrosis, fibrosis improvement was observed in five of six patients; however, given the small sample size, absence of a control group, and exploratory endpoints, these findings represent early signals that require validation in adequately powered randomized controlled trials. We further address unresolved controversies, including the dual hepatoprotective versus pro-steatotic functions of ROCK and the dose-dependent bidirectional regulation of HSCs migration, which challenge systemic inhibition strategies. Looking forward, we spotlight emerging frontiers such as cell-specific nanodelivery systems (vitamin A-liposomes, M6PHSA), PROTAC-mediated ROCK degradation, non-coding RNA therapeutics, and synergistic combinations with FXR, PPAR, and GLP-1 receptor agonists. This review provides a comprehensive blueprint for translating ROCK isoform biology into precision antifibrotic therapies.

## 1 Introduction

Liver fibrosis is a pathological process of dysregulated wound-healing responses caused by various chronic liver injuries, characterized by excessive deposition of extracellular matrix (ECM) and progressive destruction of hepatic architecture. This process can further progress to cirrhosis, portal hypertension, and even hepatocellular carcinoma, imposing a substantial global health burden. Metabolic dysfunction-associated steatotic liver disease (MASLD) and its more severe form, metabolic dysfunction-associated steatohepatitis (MASH), have become one of the leading etiologies of liver fibrosis, affecting approximately 25% of the global population (Sousa-Lima et al., 2021). Although progress has been made in elucidating the pathogenesis of liver fibrosis, effective antifibrotic treatments remain scarce. There is an urgent need to deepen the understanding of its molecular regulatory network to identify novel therapeutic targets.

Rho-associated coiled-coil containing protein kinase (ROCK) belongs to the serine/threonine protein kinase family and serves as the major downstream effector of Rho family small GTPases (RhoA, RhoB, RhoC). ROCK exists in two isoforms, ROCK1 and ROCK2, which are widely expressed in multiple tissues and regulate a broad spectrum of cellular functions, including cell contraction, migration, proliferation, apoptosis, and gene expression (Sousa-Limaet al., 2021). In the liver, aberrant activation of the ROCK signaling pathway is closely associated with the development and progression of liver fibrosis, and plays a particularly central role in hepatic stellate cells (HSCs) activation, contraction, and ECM production (Iwamoto et al., 2000).

In recent years, extensive studies have revealed the multifaceted mechanisms of the Rho/ROCK signaling pathway in liver fibrosis. This pathway not only directly regulates cytoskeletal reorganization and contractile function of HSCs, but also modulates hepatic inflammatory responses, immune cell functions, and phenotypic transition of liver sinusoidal endothelial cells (LSECs) through crosstalk with other key signaling pathways such as transforming growth factor-β (TGF-β)/suppressor of mother against decapentaplegic (Smad), Wingless-Int1 (Wnt)/β-catenin, phosphoinositide 3-kinase (PI3K)/protein kinase B (AKT), and mitogen-activated protein kinases (MAPK) (Xu et al., 2018; Nakhaei-Rad et al., 2025). In addition, different ROCK isoforms exhibit differential functions during liver fibrosis: ROCK1 seems mainly involved in hepatocyte lipid metabolism disorders and macrophage inflammatory responses, whereas ROCK2 appears to play a more prominent role in HSCs activation and vascular microenvironment regulation (Sousa-Lima et al., 2021; Hu et al., 2026). Recent comprehensive reviews have systematically summarized the landscape of anti-fibrotic therapies for MASH (Tacke et al., 2023; Schwabe et al., 2025), highlighting the growing recognition of the Rho/ROCK pathway as a promising therapeutic target in this field.

Given the pivotal role of the Rho/ROCK pathway in liver fibrosis, targeted therapeutic strategies against this pathway have become a research hotspot in antifibrotic drug development. A variety of ROCK inhibitors, including non-selective agents (e.g., fasudil, Y-27632) and selective ROCK2 inhibitors (e.g., KD025, GV101, TDI01), have shown antifibrotic activity in preclinical models (Zanin-Zhorov et al., 2023; Han et al., 2021; Tada et al., 2001). Among them, the selective ROCK2 inhibitor TDI01 has completed Phase I clinical trials and initially showed a trend toward fibrosis reduction in patients with liver fibrosis, marking an important advance in the translation of this field from basic research to clinical application (Hu et al., 2026).

This review aims to systematically elaborate on the roles and mechanisms of the Rho/ROCK signaling pathway in liver fibrosis, with a focus on its core function in HSCs activation and contraction, its differential roles in various liver cell types, its interactive regulatory network with other signaling pathways, and its latest advances in antifibrotic therapeutic strategies targeting this pathway, so as to provide a comprehensive perspective for mechanistic research and clinical management of liver fibrosis.

## 2 Molecular structure, isoforms, and signal transduction regulation of ROCK

### 2.1 Molecular structure and classical signaling of ROCK isoforms

ROCK belongs to the serine/threonine protein kinase family, and mammals express two isoforms: ROCK1 and ROCK2. The two share high structural similarity (approximately 65% homology), both containing an N-terminal kinase domain, a central coiled-coil domain, a C-terminal pleckstrin homology domain, and a cysteine-rich region (Matoba et al., 2020; Shimizu and Liao, 2016). ROCK activation is mainly dependent on upstream GTP-bound RhoA, RhoB, or RhoC, which interact with the coiled-coil region of ROCK to relieve its autoinhibitory state (Shimizu and Liao, 2016; Hartmann et al., 2015).

Despite structural similarity, ROCK1 and ROCK2 differ remarkably in function. During epithelial-mesenchymal transition (EMT) in renal proximal tubular epithelial cells, RhoA inhibits E-cadherin transcription by upregulating Snail1, whereas RhoC promotes proteasomal degradation of E-cadherin (Hutchison et al., 2009). Upregulation of α-smooth muscle actin (α-SMA) can be blocked by RhoA knockdown but is not affected by RhoB or RhoC silencing; stress fiber formation is completely dependent on RhoC (Hutchison et al., 2009).

Downstream substrates of ROCK are extensively involved in cytoskeletal remodeling and contraction regulation, among which the most classic are the myosin-binding subunit (MYPT1) of myosin light chain phosphatase (MLCP) and myosin light chain (MLC) (Shimizu and Liao, 2016). ROCK inhibits MLCP activity by phosphorylating MYPT1, and simultaneously directly phosphorylates MLC; these two phosphorylation events act synergistically to promote stress fiber formation (Hartmann et al., 2015). In addition, ROCK can phosphorylate LIM kinase (LIMK), which further inactivates cofilin to stabilize actin filaments (Shimizu and Liao, 2016). These effects collectively constitute the molecular basis for ROCK-mediated regulation of cell migration, adhesion, proliferation, and phenotypic transformation (Matoba et al., 2020).

### 2.2 ROCK isoform activity assessment: beyond expression levels

A critical consideration in interpreting existing studies is the distinction between ROCK isoform expression and actual kinase activity. Elevated mRNA or protein levels of a given ROCK isoform do not equate to heightened pathway output, because catalytic activity is contingent on upstream Rho–GTP loading, post-translational modifications, and subcellular localization.

The most commonly employed surrogate markers are phosphorylation-based readouts—p-MLC (Ser19), p-MYPT1 (Thr696/Thr853), and p-LIMK/p-cofilin (Ser3)—which reflect downstream functional output. More definitive evidence comes from direct kinase activity assays, such as *in vitro* phosphorylation of recombinant MYPT1 by immunoprecipitated ROCK (Dohnalkova et al., 2023), and FRET-based biosensors that enable real-time monitoring of isoform-specific catalytic activity in living cells.

Notably, transcriptomic approaches, including single-cell RNA sequencing, can delineate isoform-specific expression patterns but remain unable to discriminate between catalytically active and inactive ROCK pools. Addressing this gap requires integrated phosphoproteomic or functional validation to confirm pathway engagement within defined cell populations.

## 3 Core role of the Rho/ROCK signaling pathway in hepatic stellate cell activation and contraction

HSCs activation is the core event in liver fibrosis. In normal liver, HSCs remain in a quiescent state, store vitamin A and maintain the hepatic sinusoidal microenvironment. Upon liver injury, HSCs are activated and transdifferentiate into myofibroblast-like cells, expressing α-SMA, producing large amounts of ECM components, and acquiring contractile capacity, which increases intrahepatic vascular resistance and drives the development of portal hypertension (Nakhaei-Rad et al., 2025; Yee, 1998). The Rho/ROCK signaling pathway plays an irreplaceable regulatory role in this process.

### 3.1 The Rho/ROCK pathway regulates morphological transformation and activation of HSCs

The transition of HSCs from a quiescent to an activated state is accompanied by marked morphological changes, including the disappearance of dendritic processes, the formation of actin stress fibers, and the increase in cell volume. Early studies have confirmed that Rho small GTPases are key molecules regulating HSCs morphological changes. Yee et al. found that specific inhibition of Rho activity using C3 transferase blocked and reversed morphological changes and stress fiber formation in activated HSCs, demonstrating that the Rho signaling pathway directly regulates activation-associated cytoskeletal reorganization (Yee, 1998). Kato et al. further confirmed that overexpression of the Rho inhibitor Rho GDP dissociation inhibitor or dominant-negative Rho mutants induced atrophic morphology and inhibited stress fiber formation in HSCs, while significantly suppressing type I collagen accumulation (Kato et al., 1999).

As a key downstream effector of Rho, ROCK plays an important role in HSCs activation. Iwamoto et al. found that the p160ROCK-specific inhibitor Y-27632 blocked HSCs spreading on fibronectin-coated dishes, inhibited RhoA-induced stress fiber formation, and reduced phosphorylation levels of focal adhesion kinase and extracellular signal-regulated kinase (Iwamoto et al., 2000). Y-27632 treatment also led to impaired HSCs proliferation, which was associated with decreased cyclin D1 expression and upregulation of the cell cycle inhibitor p27. Furthermore, Y-27632 significantly reduced gene expression and protein accumulation of type I collagen, suggesting that ROCK-mediated stress fiber assembly is a key pathological link in liver fibrosis (Iwamoto et al., 2000). Beyond classical biochemical stimuli, mechanical forces also activate the Rho/ROCK pathway in HSCs: Huang et al. demonstrated that hydrostatic pressure (50 mmHg) induces profibrotic properties in HSCs via the RhoA/ROCK signaling pathway, significantly upregulating α-SMA, TGF-β1, and p-MLC expression, all effectively blocked by Y-27632 (Huang et al., 2022). This mechanical regulation is further contextualized by integrin-mediated mechanosignaling, where the RhoA/ROCK pathway acts as a key downstream effector linking extracellular matrix mechanical properties to HSC activation (Sharip and Kunz, 2025).

Notably, recent evidence has linked ROCK1 to ferroptosis regulation in HSCs. Wang et al. demonstrated that artesunate promotes the ubiquitination and degradation of ROCK1, thereby relieving its phosphorylation of ATF3 and subsequently enhancing the nuclear accumulation of ATF3, which ultimately triggers ferroptosis in hepatic stellate cells (HSCs) (Wang et al., 2024). Notably, knockdown of Atf3 or overexpression of Rock1 was able to reverse this effect. This finding represents the first connection between ROCK isoform signaling and ferroptosis in liver fibrosis (Wang et al., 2024).

Ras-related C3 botulinum toxin substrate 1 (Rac1), another important member of the Rho family, also exerts profibrotic effects in HSCs. Choi et al. used a transgenic mouse model with α-SMA promoter-driven constitutively active Rac1 and found that sustained Rac1 activation promoted reactive oxygen species (ROS) production and enhanced expression of activation markers and collagen in HSCs (Choi et al., 2006). In a carbon tetrachloride (CCl_4_)-induced liver injury model, Rac1 transgenic mice exhibited more severe liver injury, hepatocyte apoptosis, and liver fibrosis (Choi et al., 2006).

### 3.2 Rho/ROCK pathway mediates HSCs contraction and portal hypertension

The contractile function of HSCs is a key determinant of intrahepatic vascular resistance and portal pressure. In fibrotic liver, activated HSCs surround the sinusoids, and their contraction leads to sinusoidal narrowing and markedly increased intrahepatic blood flow resistance, thereby contributing to the development of portal hypertension (Xu et al., 2018; Iizuka et al., 2011). The Rho/ROCK pathway is the principal signaling mechanism for HSCs contraction, which is achieved by regulating MLC phosphorylation levels.

Kawada et al. first discovered that Y-27632 significantly inhibited endothelin-1 (ET-1)-induced HSCs contraction, and reduced F-actin stress fiber formation and MLC phosphorylation (Kawada et al., 1999). Isolated liver perfusion experiments further confirmed that Y-27632 almost completely abolished ET-1-induced portal pressure elevation while ameliorating hepatocyte degeneration (Kawada et al., 1999).

The molecular mechanism of HSCs contraction involves both Ca^2+^-dependent and Ca^2+^-independent pathways. Iizuka et al. found that during HSCs activation, the expression of MLC kinase (MLCK), ROCK2, and protein kinase C (PKC) substrate CPI-17 was all significantly upregulated (Iizuka et al., 2011). Upon ET-1 stimulation, PKC-mediated CPI-17 phosphorylation and ROCK-mediated MYPT1 phosphorylation jointly promote sustained MLC phosphorylation, thereby enhancing HSCs contractility. Melton et al. directly measured HSCs contractile force and found that even after complete chelation of intracellular Ca^2+^, ET-1 still induced significant contractile force and MLC phosphorylation, whereas Y-27632 inhibited more than 70% of the contractile force, indicating that the Ca^2+^-independent Rho/ROCK pathway is the dominant mechanism of HSCs contraction (Melton et al., 2006).

Multiple vasoactive factors regulate HSCs contraction through the Rho/ROCK pathway. Zhang et al. found that angiotensin II (Ang II) activates the Rho/ROCK pathway via the angiotensin type 1 receptor (AT1R), promoting MLC phosphorylation and HSCs contraction (Zhang et al., 2008a; Zhang et al., 2008b). Aldosterone induces HSCs contraction through mineralocorticoid receptor-mediated activation of the RhoA/ROCK-2/p-MLC cascade (Ji et al., 2011). ET-1, the most potent HSCs contraction agonist, exerts its effects mainly through the Rho/ROCK pathway, and Y-27632 can effectively inhibit HSCs contraction even in the presence of ET-1 (Kawada et al., 1999; Saab et al., 2002).

### 3.3 The Rho/ROCK pathway regulates migration and proliferation of HSCs

During liver fibrosis, migration of activated HSCs to injury sites is a critical step in fibrosis progression. The Rho/ROCK pathway plays a dual role in regulating HSCs migration. Li et al. found that platelet-derived growth factor-BB (PDGF-BB) induced HSCs cytoskeletal reorganization and promoted migration by activating RhoA, but both constitutively active RhoA and dominant-negative RhoA inhibited migration, suggesting that RhoA activity must be maintained at an appropriate level to support cell migration (Li et al., 2010).

The effect of ROCK inhibitors on HSCs migration is concentration-dependent. Yanase et al. reported that low-concentration Y-27632 (10^−7^ M) inhibited HSCs migration, whereas high-concentration (10^−4^ M) enhanced migration, which may be related to differences in cytoskeletal reorganization patterns at different concentrations (Yanase et al., 2003). A study by Bachtler et al. confirmed that Y-27632 and Y-33075 increased cell migration while inhibiting HSCs contraction, suggesting that ROCK inhibition may promote HSCs motility by relieving negative regulation of migration (Bachtler et al., 2023).

HSCs proliferation is also regulated by the Rho/ROCK pathway. Fasudil inhibits HSCs proliferation by suppressing ROCK activity, and the mechanism involves inhibition of the MAPK signaling pathway (Fukushima et al., 2005). Y-27632 blocks HSCs cell cycle progression by regulating the expression of cyclin D1 and p27 (Iwamoto et al., 2000). In addition, inhibition of Rho or ROCK activity can induce HSCs apoptosis, whereas lysophosphatidic acid inhibits HSCs apoptosis by activating the Rho/ROCK pathway (Ikeda et al., 2003).

## 4 Differential functions of the Rho/ROCK pathway in liver fibrosis-associated cell types

The role of the Rho/ROCK signaling pathway in liver fibrosis is not limited to HSCs, but involves multiple intrahepatic cell types, including LSECs, hepatocytes, macrophages, and immune cells. The differential expression and function of ROCK isoforms in different cell types provide an important basis for precision therapy of liver fibrosis.

### 4.1 ROCK2 in liver sinusoidal endothelial cells and vascular microenvironment regulation

LSECs are specialized endothelial cells that form the sinusoidal wall, and maintenance of their phenotype and function is critical for liver homeostasis. During liver fibrosis, LSECs undergo capillarization, losing their fenestrated structure and secreting profibrotic factors, thus forming a profibrotic vascular microenvironment. The interplay between actin and MLC, regulated by ROCK-mediated MLC phosphorylation, has been shown to control fenestration size and number in LSECs, providing a mechanical framework for understanding sinusoidal capillarization (Zapotoczny et al., 2022).

A recent study by Hu et al. revealed the critical role of ROCK2 in LSECs: ROCK2 expression was significantly elevated in LSECs and perivascular HSCs of patients with liver fibrosis (Hu et al., 2026). Importantly, this study also demonstrated increased ROCK2 activity as evidenced by elevated p-MYPT1 levels detected by immunohistochemistry in fibrotic liver tissues, supporting a functional role for ROCK2 activation in this context. However, it should be noted that p-MYPT1 is not exclusively specific to ROCK2 and may also reflect ROCK1 activity; isoform-specific activity assessment in LSECs awaits the development of more selective biosensors. Nevertheless, through single-cell analysis, gene knockout models, and proteomic studies, the team confirmed that ROCK2-driven endothelial cytoskeletal alterations trigger profibrotic angiocrine signaling, which in turn activates perivascular HSCs (Hu et al., 2026). The significance of this discovery is underscored by accompanying commentaries in Cell and Nature Reviews Drug Discovery, which emphasized that ROCK2 represents a druggable angiocrine target in liver fibrosis and highlighted the translational potential from mechanism to early clinical application (Cao and Shah, 2026; Leake, 2026).

Based on this finding, the researchers developed the highly selective ROCK2 inhibitor TDI01. In rodent and minipig MASH models, TDI01 restored the normal phenotype of LSECs and attenuated liver fibrosis (Hu et al., 2026). A Phase I clinical trial (ChiCTR2200058868) of TDI01 in 62 healthy volunteers demonstrated favorable pharmacokinetics (double-peak phenomenon attributable to hepato-enteral circulation) and a good safety profile with no severe adverse events (Hu et al., 2026). An extended, single-arm, open-label study (ChiCTR2400082056) enrolled six patients with liver fibrosis who received TDI01 at 200 mg once daily for 24 weeks (Hu et al., 2026). Efficacy was assessed by liver stiffness measurement (LSM) using transient elastography (FibroScan) and serum fibrosis biomarkers. Five of six patients showed a trend toward sustained reductions in LSM and improvements in fibrosis-related biomarkers; liver biopsies in available patients suggested reduced collagen deposition, including in one patient with F4 cirrhosis (Hu et al., 2026). However, given the small sample size (n = 6), the absence of a control group, the exploratory endpoints, and the use of descriptive statistics without formal hypothesis testing, these observations should be interpreted as preliminary signals rather than definitive evidence of antifibrotic efficacy. Rigorous randomized controlled trials with larger sample sizes, predefined histological endpoints (e.g., ≥1 stage fibrosis improvement by Ishak score), and adequate treatment duration are needed to establish the clinical antifibrotic efficacy of TDI01 (Hu et al., 2026; Sun et al., 2026).

### 4.2 ROCK signaling in hepatocytes and lipid metabolism and cell survival

ROCK signaling in hepatocytes plays complex and sometimes seemingly contradictory roles in liver fibrosis. In a complementary study, Tak et al. revealed that ER stress-induced Gα12 overexpression in hepatocytes activates ROCK1, which downregulates miR-15a and upregulates ALOX12, promoting ferroptosis and acute liver injury; ROCK1 inhibition effectively rescued hepatotoxic liver damage, establishing a Gα12/ER stress/ROCK1/ferroptosis axis (Tak et al., 2022). In the liver of patients with MASH, ROCK1 expression is significantly elevated, and hepatocyte-specific ROCK1 knockout attenuates diet-induced hepatic steatosis, cell death, inflammation, and fibrosis in mice through a mechanism associated with reduced myeloid cell recruitment, whereas myeloid cell-specific ROCK1 deletion did not show a protective effect (Dohnalkova et al., 2023).

However, hepatocyte ROCK signaling plays a critical role in protecting hepatocytes from injury. Herrera et al. used a conditional gene knockout mouse model and found that hepatocytes lacking both ROCK1 and ROCK2 exhibited widespread early death, aggravated hepatocyte injury, and decreased liver function after 3,5-diethoxycarbonyl-1,4-dihydrocollidine diet-induced liver injury (Herrera et al., 2025). RNA-seq analysis showed that cell death, inflammation, and profibrotic pathways were significantly upregulated in double-knockout livers, and the p21-mediated caspase-3-dependent apoptotic pathway was activated (Herrera et al., 2025). These findings reveal the protective role of ROCK signaling in hepatocyte survival, suggesting that systemic ROCK inhibition may carry the risk of hepatocyte injury.

The apparently contradictory roles of ROCK signaling in hepatocytes, promoting steatosis in metabolic contexts while protecting against acute injury, may be reconciled by considering several factors. First, the studies revealing protective roles used combined ROCK1/ROCK2 deletion (Herrera et al., 2025), which may have effects distinct from selective ROCK1 inhibition. It is possible that ROCK2 has a more prominent protective role in hepatocyte survival, while ROCK1 is more specifically involved in metabolic dysregulation. This interpretation is consistent with the differential substrate specificity of the two isoforms.

Second, the temporal dimension may be critical: acute ROCK activation may serve protective functions (e.g., maintaining cell polarity, regulating tight junctions, mediating cytoprotective autophagy), whereas chronic ROCK activation in the setting of metabolic disease may drive pathological remodeling. This is analogous to the well-known paradox of TGF-β signaling in liver fibrosis, where acute signaling promotes hepatocyte regeneration while chronic signaling drives fibrogenesis.

Third, the level of ROCK activity may determine the functional outcome. Excessive ROCK activity is clearly pathological, but complete absence of ROCK signaling may also be detrimental, as ROCK1/2 are essential for fundamental cellular processes including cytokinesis, cell adhesion, and migration. This biphasic relationship poses a significant challenge for therapeutic targeting.

To experimentally resolve this paradox, we propose the following approaches: (i) generation of inducible, hepatocyte-specific single knockouts to distinguish isoform-specific functions; (ii) acute vs. chronic ROCK inhibition studies using dose-titratable inhibitors or temporally controlled genetic systems (e.g., tamoxifen-inducible Cre); (iii) phosphoproteomic profiling of hepatocytes under metabolic stress (e.g., palmitate/oleate treatment) vs. acute injury (e.g., CCl_4_) to map isoform-specific substrate engagement; and (iv) single-cell multi-omics (scRNA-seq + CITE-seq for phospho-epitopes) in human liver biopsies from MASLD vs. acute liver failure patients to compare ROCK activity states in hepatocytes.

### 4.3 ROCK signaling in macrophages and immune cells and inflammation regulation

Hepatic macrophages (Kupffer cells) play a key role in the initiation and progression of liver fibrosis. ROCK signaling in macrophages regulates inflammatory cytokine production, cell migration, and polarization direction. Nalkurthi et al. found that the selective ROCK2 inhibitor KD025 effectively attenuated liver fibrosis and promoted fibrosis resolution in a thioacetamide (TAA)-induced liver fibrosis model (Nalkurthi et al., 2022). Mechanistically, KD025 inhibited tumor necrosis factor α (TNF-α) production by hepatic macrophages, disrupted macrophage niches within fibrotic septa, and directly regulated macrophage function by interfering with the Signal transducer and activator of transcription 3 (STAT3)/Cofilin signaling pathway (Nalkurthi et al., 2022).

ROCK1 also plays an important role in macrophage-mediated liver fibrosis. Wei et al. found that receptor-interacting protein 3 (RIP3) was upregulated in liver tissues and macrophages of patients with liver fibrosis and mouse models (Wei et al., 2019). Macrophage-specific RIP3 deficiency attenuated CCl_4_-or bile duct ligation (BDL)-induced liver inflammation and fibrosis. Mechanistically, RIP3 alleviates liver fibrosis by inhibiting the ROCK1-Toll-like receptor 4 (TLR4)-nuclear factor-κB (NF-κB) pathway, and ROCK1 overexpression can reverse the protective effect of RIP3 deficiency (Wei et al., 2019).

Th17 cells are an important immune cell subset involved in liver fibrosis. Zhou et al. found that fasudil downregulated Th17 cell differentiation and effector cytokine secretion while upregulating Treg cells by inhibiting the Rho/ROCK pathway, thereby attenuating liver granuloma and fibrosis induced by *Schistosoma japonicum* infection (Zhou et al., 2019). The ROCK2 inhibitor KD025 also significantly reduced hepatic interleukin-17 (IL-17) levels and the number of splenic germinal centers (Nalkurthi et al., 2022).

### 4.4 Differential functions of ROCK1 and ROCK2 in liver fibrosis

Current evidence for isoform-specific functions in liver fibrosis comes from multiple sources with varying degrees of rigor. We therefore distinguish three categories of evidence throughout Section 4: (i) genetic evidence, derived from isoform-specific conditional knockout mice (e.g., ROCK1^Δhep^, ROCK2^ΔEC^) or isoform-specific siRNA/shRNA knockdown; (ii) pharmacological evidence, derived from selective inhibitors such as KD025 (ROCK2-selective), GV101 (ROCK2-selective, >1000-fold), and TDI01 (ROCK2-selective); and (iii) expression-based or activation-based evidence, derived from transcriptomic data (including scRNA-seq), immunohistochemistry, Western blot, or phosphorylation assays (e.g., p-MYPT1, p-MLC, p-cofilin) (Table 1). Conclusions derived from pan-ROCK inhibitors (e.g., Y-27632, fasudil) or transcriptomic data alone should be interpreted with caution, as they do not directly establish isoform-specific causality.

**TABLE 1:** Summary of ROCK1 and ROCK2 functions in hepatic cell types: evidence levels and key findings.

Cell type	ROCK isoform	Reported function	Level of evidence	Key References	Caveats and gaps
HSCs	ROCK1	Contraction (ET-1, Ang II); migration (PDGF-BB); activation (TGF-β1)	Genetic (siRNA), Pharmacological (Y-27632, fasudil), Activation (p-MLC)	(Iwamoto et al., 2000; Kawada et al., 1999; Li et al., 2010; Shimada et al., 2011)	Evidence from pan-ROCK inhibitors; isoform-specific knockout (KO) not available for HSC contraction
HSCs	ROCK2	Contraction; cortical actin; profibrogenic signaling via p-cofilin	Genetic (siRNA), Pharmacological (KD025, GV101, TDI01), Activation (p-cofilin, p-MYPT1)	(Ji et al., 2011; Hu et al., 2026; Zanin-Zhorov et al., 2023; Nalkurthi et al., 2022)	More selective evidence but still largely from pharmacological studies
LSECs	ROCK1	Widely expressed; less cell-type-specific data	Expression-based (scRNA-seq)	(Hu et al., 2026)	scRNA-seq shows broad distribution; no isoform-specific KO data in LSECs
LSECs	ROCK2	Selectively upregulated in fibrotic LSECs; drives angiocrine profibrotic signaling; capillarization	Genetic (endothelial cell-specific KO), Activation (p-MYPT1), Pharmacological (TDI01)	(Hu et al., 2026)	Landmark study but single-group; p-MYPT1 not exclusively ROCK2-specific
Hepatocytes	ROCK1	Promotes steatosis, lipotoxicity, inflammation in NASH	Genetic (hepatocyte-specific KO), Direct kinase activity	(Dohnalkova et al., 2023)	Strong genetic evidence; ROCK1^Δhep^ may not distinguish direct vs. paracrine effects
Hepatocytes	ROCK1/2 (combined)	Protective against acute injury (porphyria-associated)	Genetic (combined ROCK1/2 KO)	(Herrera et al., 2025)	Combined KO does not distinguish individual isoform contributions
Macrophages	ROCK1	Promotes inflammatory polarization; ROCK1-TLR4-NF-κB axis	Genetic (siRNA), Pharmacological (Y-27632)	(Wei et al., 2019; Chong et al., 2022)	Evidence from pan-ROCK inhibitors; ROCK1-specific macrophage KO lacking
Macrophages	ROCK2	Regulates profibrogenic macrophage differentiation; p-STAT3/cofilin	Pharmacological (KD025, GV101), Activation (p-cofilin, p-STAT3)	(Nalkurthi et al., 2022; Zanin-Zhorov et al., 2023)	Pharmacological evidence strong; isoform-specific macrophage KO needed
T cells	ROCK2	Th17/Treg balance regulation; IL-17/IL-21 secretion	Genetic (T cell-specific KO), Pharmacological (KD025)	(Zhou et al., 2019)	Mainly from cGVHD models; liver-specific data limited

With this evidence framework in mind, we briefly summarize the best-supported isoform-specific findings across major hepatic cell types. Although ROCK1 and ROCK2 are highly homologous, they exhibit differential functions in liver fibrosis. Zanin-Zhorov A et al. compared the effects of the selective ROCK2 inhibitor GV101 with those of non-selective ROCK inhibitors and found that GV101 significantly reduced established liver fibrosis in a TAA-combined with a high-fat diet-induced liver fibrosis model, decreased hepatic collagen levels, downregulated pCofilin, pSTAT3, and pAkt, and simultaneously upregulated pSTAT5 and pAMPK (Zanin-Zhorov et al., 2023). *In vitro*, GV101 inhibited profibrotic marker expression in fibroblasts, adipocyte differentiation, and TLR-induced cytokine secretion by innate immune cells (Zanin-Zhorov et al., 2023).

In HSCs, ROCK2 appears to be the main isoform regulating contraction. Shimada et al. found via siRNA knockdown that ROCK1 mainly mediates ET-1 synthesis, whereas both ROCK1 and ROCK2 are involved in TGF-β2 synthesis (Shimada et al., 2011). During HSCs activation, the expression of ROCK2 is more significantly upregulated (Iizuka et al., 2011). However, in macrophages, ROCK1 seems to play a predominant role in regulating the TLR4-NF-κB pathway (Wei et al., 2019). These isoform-specific functions provide a theoretical basis for the development of selective ROCK inhibitors to achieve precision therapy.

In conclusion, the Rho/ROCK signaling pathway exerts multi-level regulatory effects in HSCs, LSECs, hepatocytes, and macrophages through the distinct functional roles of its two subtypes, ROCK1 and ROCK2 (Figure 1).

**FIGURE 1:**
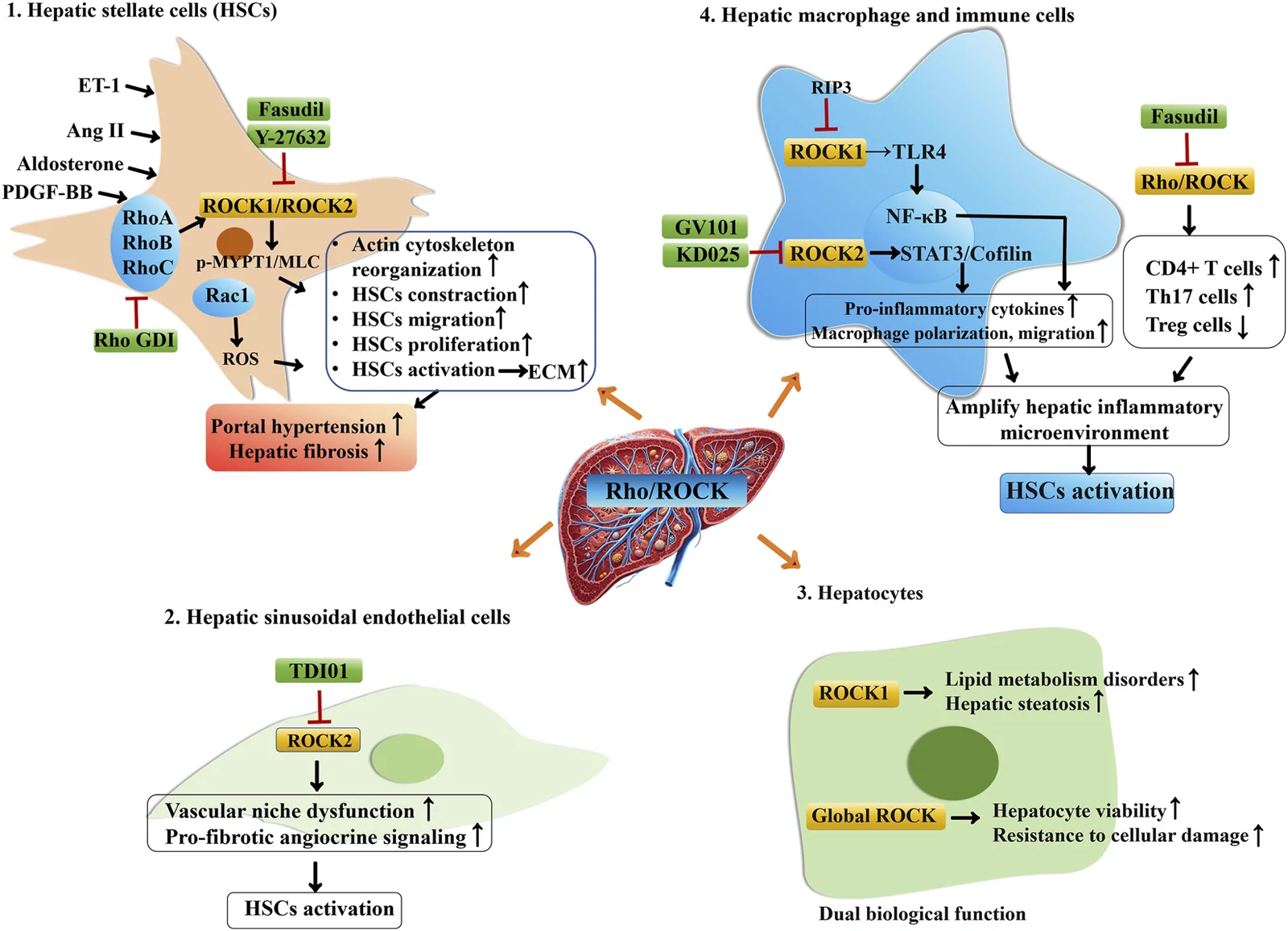
The central role of the Rho/ROCK signaling pathway in liver fibrosis and its cell-type-specific regulation. The Rho/ROCK pathway drives HSCs activation, contraction, and ECM production by regulating MLC phosphorylation and cytoskeletal reorganization, constituting a central signaling hub in liver fibrosis. The two isoforms of this pathway exhibit cell- and context-dependent contributions: ROCK2 appears to play a predominant role in several contexts, particularly in HSC contraction, LSEC capillarization, and Th17 cell polarization, whereas ROCK1 is more specifically involved in hepatocyte lipid metabolism and macrophage inflammatory responses; however, the contributions of both isoforms are context-dependent. ECM: extracellular matrix; ROCK: Rho-associated coiled-coil containing protein kinase; MLC: myosin light chain; MYPT1: myosin-binding subunit of myosin light chain phosphatase.

## 5 Interactive regulatory network between Rho/ROCK signaling and other key pathways

The Rho/ROCK signaling pathway forms a complex interactive regulatory network with multiple signaling pathways, which collectively determine the progression of liver fibrosis.

### 5.1 Interaction between Rho/ROCK and TGF-β/Smad pathway

TGF-β is the most potent profibrotic cytokine in liver fibrosis. Shimada et al. found that in TGF-β1-stimulated human HSCs, ROCK activation is not only involved in the Smad pathway but also independently regulates the expression of profibrotic factors (Shimada et al., 2011). ROCK inhibitors significantly inhibited TGF-β1-induced synthesis of ET-1 and TGF-β2, and ET-1 synthesis was mainly mediated by ROCK1 (Shimada et al., 2011).

Cho et al. revealed the mechanism by which E-cadherin (ECAD) antagonizes TGF-β1 gene induction by inhibiting RhoA-dependent Smad3 phosphorylation (Cho et al., 2010). The p120-catenin binding domain of ECAD is an essential structural domain for this inhibitory effect. ECAD binds p120-catenin and recruits RhoA, preventing TGF-β1 from enhancing RhoA-mediated Smad3 phosphorylation (Cho et al., 2010).

### 5.2 Interaction between Rho/ROCK and Wnt/β-catenin pathway

Zhang et al. found that Wnt/β-catenin signaling is involved in the development of portal hypertension by regulating HSCs contraction (Zhang et al., 2021). Wnt3a stimulation induces Smo-independent glioma-associated oncogene 1 (Gli1) nuclear translocation, which subsequently leads to HSCs contraction through leukemia-associated RhoGEF-mediated RhoA activation. Mechanistically, Wnt/β-catenin transcriptionally represses the expression of suppressor of fused homolog (Sufu) through β-catenin/Transcription Factor 4 (TCF4) interaction and TCF4 binding to the Sufu promoter, thereby relieving the inhibition of Gli1 nuclear translocation (Zhang et al., 2021).

### 5.3 Interaction between Rho/ROCK and Hippo/YAP pathway

Park et al. revealed the mechanism by which sphingosine-1-phosphate (S1P)/sphingosine-1-phosphate receptor 2 (S1PR2) activates yes-associated protein (YAP) through the RhoA/ROCK pathway (Park et al., 2024). Exogenous S1P activates the proto-oncogene tyrosine-protein kinase Src-RhoA-ROCK signaling axis via S1P receptor 2, inhibits the Hippo kinase cascade, thereby activating YAP and upregulating the expression of connective tissue growth factor (CTGF), collagen type I alpha one chain (COL1A1), and α-SMA. Zhang et al. found that Ang II-induced HSCs contraction promotes YAP nuclear translocation and COL1A1 expression through the Rho/ROCK pathway, suggesting that mechanical signals regulate ECM production via YAP (Zhang et al., 2024).

### 5.4 Interaction between Rho/ROCK and NOX4/ROS pathway

In HSC-T6 cells, the NADPH oxidase 4 (NOX4)/ROS pathway lies upstream of the RhoA/ROCK1 pathway and positively regulates its activity, but there is no direct interaction between NOX4 and RhoA protein (Huang et al., 2019). Ursolic acid can reverse liver fibrosis by simultaneously inhibiting the NOX4/ROS and RhoA/ROCK1 pathways, and there is interactive regulation between these two pathways (Wan et al., 2020).

### 5.5 Interaction between Rho/ROCK and PI3K/Akt and MAPK pathway

The ROCK inhibitor HA-1077 protects hepatocytes from CCl_4_-induced acute liver injury by activating the PI3K/Akt pathway (Ikeda et al., 2007). Fasudil inhibits phosphorylation of extracellular signal-regulated kinase 1/2 (ERK1/2), c-Jun N-terminal kinase and p38 MAPK in HSCs (Fukushima et al., 2005). Soluble antigens of *Echinococcus multilocularis* induce M2 macrophage polarization by activating the RhoA-MAPK pathway, thereby promoting HSCs activation and liver fibrosis (Chong et al., 2022).

### 5.6 Interaction between Rho/ROCK and cAMP/PKA pathway

The cyclic adenosine monophosphate (cAMP)/protein kinase A (PKA) signaling pathway generally exerts antifibrotic effects. Elnagdy et al. demonstrated that the cAMP/PKA pathway intersects with ROCK signaling in the regulation of HSCs migration (Elnagdy et al., 2023). Zeng et al. found that the Dantao formula may alleviate liver fibrosis and portal hypertension by regulating the cAMP/PKA/ROCK signaling pathway (Zeng et al., 2026). The mechanism involves upregulation of cAMP and PKA, and downregulation of ROCK and p-MLC (Zeng et al., 2026).

## 6 Antifibrotic therapeutic strategies targeting the Rho/ROCK signaling pathway and advances

Given the central role of the Rho/ROCK pathway in liver fibrosis, targeted therapeutic strategies against this pathway have become a hotspot in antifibrotic drug research and development. From non-selective ROCK inhibitors to selective ROCK2 inhibitors and then to targeted drug delivery systems, significant progress has been made in this field.

### 6.1 Non-selective ROCK inhibitors: fasudil and Y-27632

Fasudil is the only clinically approved ROCK inhibitor to date, mainly used to improve cerebral microcirculation. In the field of liver fibrosis, fasudil attenuates TAA-induced liver fibrosis by activating natural killer (NK) cells and inhibiting HSCs activation, reduces the expression of α-SMA, matrix metallopeptidase (MMP)-2, MMP-9, and TGF-β1, promotes HSCs apoptosis and inhibits its proliferation (Han et al., 2021). Fasudil also shows protective effects in diabetes-associated liver fibrosis (Zhou et al., 2014; Xie et al., 2018).

Y-27632 is the most commonly used experimental ROCK inhibitor. Murata et al. found that Y-27632 inhibited the expression of the Na^+^/Ca^2+^ exchanger, an activation marker of HSCs, reduced the number of α-SMA- and TGF-β1-positive cells and decreased hepatic hydroxyproline content, thereby attenuating CCl_4_-induced liver fibrosis (Murata et al., 2003; Murata et al., 2001).

Y-27632 also shows protective effects in hepatic ischemia-reperfusion injury, inhibiting HSCs contraction and improving sinusoidal microcirculation (Mizunuma et al., 2002; Mizunuma et al., 2003). In fatty liver models, Y-27632 also attenuates ischemia-reperfusion injury (Kuroda et al., 2012).

However, non-selective ROCK inhibitors have significant systemic adverse effects, particularly hypotension. Klein et al. confirmed that although systemic ROCK inhibition reduces portal pressure, it simultaneously causes a decrease in arterial blood pressure, limiting its clinical application (Klein et al., 2012).

### 6.2 Selective ROCK2 inhibitors: KD025, GV101, and TDI01

Since ROCK2 appears to play a predominant role in HSCs activation and vascular microenvironment regulation, selective ROCK2 inhibitors have become a more promising therapeutic strategy.

KD025 (SLx-2119) is the first selective ROCK2 inhibitor to enter clinical trials. Nalkurthi et al. confirmed in a TAA-induced liver fibrosis model that both prophylactic and therapeutic administration of KD025 effectively attenuated liver fibrosis, through mechanisms involving inhibition of macrophage TNF-α production, disruption of macrophage niches and regulation of the STAT3/Cofilin signaling pathway (Nalkurthi et al., 2022).

GV101 is another highly selective ROCK2 inhibitor (with >1000-fold selectivity over ROCK1). As detailed in Section 4.4, Zanin-Zhorov A et al. demonstrated that GV101 significantly reduced established liver fibrosis in a TAA-combined, high-fat diet-induced liver fibrosis model by downregulating pCofilin, pSTAT3, and pAkt and simultaneously upregulating pSTAT5 and pAMPK (Zanin-Zhorov et al., 2023). These findings further support the therapeutic potential of selective ROCK2 inhibition beyond KD025. Recent comprehensive reviews have positioned ROCK inhibitors, particularly selective ROCK2 inhibitors, within the broader landscape of anti-fibrotic therapies for MASH (Tacke et al., 2023; Schwabe et al., 2025), underscoring their therapeutic potential.

TDI01 is a recently developed selective ROCK2 inhibitor designed to target endothelial ROCK2 in the fibrotic vascular niche. Section 4.1 comprehensively describes its preclinical pharmacology, pharmacokinetic profile, and Phase I clinical data (Hu et al., 2026). In brief, an extended open-label study in six patients with liver fibrosis showed preliminary signals of fibrosis improvement; however, adequately powered randomized controlled trials with predefined histological endpoints are needed before definitive efficacy conclusions can be drawn.

### 6.3 Targeted drug delivery systems: Improving efficacy and reducing systemic toxicity

To address the systemic adverse effects of ROCK inhibitors, various targeted drug delivery systems have been developed to specifically deliver ROCK inhibitors to HSCs.

Vitamin A-coupled liposomes enable enrichment of ROCK inhibitors in HSCs, achieving the same antifibrotic effect as free drugs at approximately 1/100 of the dose, while avoiding systemic adverse effects such as renal function decline (Okimoto et al., 2019). Kuroda et al. applied vitamin A-coupled liposome-encapsulated Y-27632 to a fatty liver ischemia-reperfusion injury model and found that targeted delivery improved survival rate, hepatic blood flow, and portal perfusion pressure without causing severe hypotension (Kuroda et al., 2015).

Mannose-6-phosphate (M6P)-modified human serum albumin (M6PHSA) can bind to the M6P/insulin-like growth factor II (IGFII) receptor, which is highly expressed on activated HSCs. van Beuge et al. conjugated Y-27632 to M6PHSA and found that the conjugate was specifically taken up by HSCs, inhibited HSCs migration and contraction, and significantly reduced collagen deposition in a CCl_4_-induced mouse model of liver fibrosis, whereas free Y-27632 was ineffective at the same dose (van Beuge et al., 2011a; van Beuge et al., 2011b).

Human serum albumin modified with a PDGFβ receptor-recognizing peptide (pPBHSA) can also target Y-27632 to activated HSCs (Klein et al., 2019). Klein et al. confirmed in cirrhotic rats that this conjugate reduced portal pressure and intrahepatic vascular resistance without affecting systemic vascular resistance (Klein et al., 2019). Notably, this targeted therapy also increased renal artery blood flow and reduced mRNA expression of α-SMA and COL1A1 in the kidney, suggesting potential additional renoprotective effects (Klein et al., 2019).

### 6.4 Regulation of the Rho/ROCK pathway by natural products and marketed drugs

Many natural products and marketed drugs exert antifibrotic effects by regulating the Rho/ROCK pathway. Ursolic acid reverses CCl_4_-induced liver fibrosis by inhibiting the NOX4/ROS and RhoA/ROCK1 signaling pathways (Wan et al., 2020). Flavonoids such as epigallocatechin-3-gallate inhibit Rho signaling in HSCs, reduce stress fiber formation and α-SMA expression, and induce HSCs apoptosis (Higashi et al., 2005). Artesunate ameliorates CCl_4_-induced hepatic fibrosis in mice by inhibiting ROCK1, which promotes the dephosphorylation and nuclear accumulation of ATF3, thereby inducing ferroptosis in HSCs (Wang et al., 2024). More recently, Panneerselvam et al. found that obtusifolin from Senna obtusifolia mitigates CCl_4_-induced liver inflammation and fibrosis by specifically modulating the ROCK2/Cofilin-1/NFκB pathway (Panneerselvam et al., 2026). Luo et al. found that Jianpi Huayu decoction alleviates hepatic fibrosis through coordinated modulation of PPARα-mediated lipid metabolic reprogramming and the ROCK pathway (Luo et al., 2026).

Statins reduce isoprenylation by inhibiting the mevalonate pathway, thereby inhibiting membrane translocation and activation of RhoA and Ras. Simvastatin attenuates liver inflammation and fibrosis in MASH mice by simultaneously inhibiting the RhoA/Rho kinase and Ras/ERK pathways (Schierwagen et al., 2016). Atorvastatin reduces portal pressure in cirrhotic rats without affecting mean arterial pressure by inhibiting intrahepatic RhoA/ROCK signaling and activating the endothelial nitric oxide synthase/PKG pathway (Trebicka et al., 2007).

Carvedilol inhibits Ang II-induced HSCs proliferation and contraction by interfering with the AT1R-mediated RhoA/ROCK2 pathway, thereby attenuating liver fibrosis (Wu et al., 2019). Spironolactone reduces portal hypertension by inhibiting ROCK2 activity and activating the nitric oxide/PKG pathway (Luo et al., 2012).

### 6.5 Regulation of the Rho/ROCK pathway by non-coding RNAs

Non-coding RNAs, especially microRNAs (miRNAs) and long non-coding RNAs (lncRNAs), play important roles in regulating the Rho/ROCK pathway.

miR-125b is upregulated during HSCs activation, enhances RhoA activity by targeting Stard13 (a RhoA-specific GTPase-activating protein), and promotes α-SMA expression and HSCs contraction. Antagonism of miR-125b significantly attenuates CCl_4_-induced liver fibrosis (You et al., 2018). In MAFLD, CpG island methylation in the miR-125b-5p promoter leads to its epigenetic silencing, which relieves the inhibition of integrin α8, activates the RhoA signaling pathway and promotes liver fibrosis (Cai et al., 2020).

miR-372-3p inhibits HSCs activation by targeting RhoC. In TGF-β1-activated LX-2 cells, miR-372-3p expression is downregulated, while RhoC expression is upregulated. Overexpression of miR-372-3p inhibits HSCs proliferation, promotes apoptosis, and reduces COL1A1 and α-SMA levels (Ou et al., 2025).

The lncRNA NORAD is upregulated in MASLD and promotes MASLD progression through two mechanisms: acting as a competing endogenous RNA to bind miR-511-3p and upregulate *Rock2* mRNA expression; and directly interacting with ROCK2 protein to inhibit its ubiquitination and degradation, thereby stabilizing ROCK2 protein (Zhang et al., 2025). Liver-specific knockdown of NORAD or overexpression of miR-511-3p can significantly slow MASLD progression (Zhang et al., 2025).

### 6.6 Synergistic strategies of ROCK inhibitors with other drugs

Given the multifactorial complexity of liver fibrosis, single-target drugs often have limited efficacy. Combination therapy with ROCK inhibitors and other antifibrotic drugs has theoretical advantages.

Combination with farnesoid X receptor (FXR) agonists: Obeticholic acid (OCA) is an approved FXR agonist that inhibits HSCs activation and bile acid synthesis (Zobair et al., 2019; Eslam et al., 2019). Preclinical studies suggest that the combination of ROCK inhibitors and OCA may produce synergistic effects through complementary mechanisms (ROCK inhibition reduces portal hypertension, and FXR agonism exerts anti-inflammatory and antifibrotic effects).

Combination with peroxisome proliferator-activated receptor (PPAR) agonists: Elafibranor (a PPARα/δ agonist) has shown antifibrotic activity in MASH clinical trials (Ratziu et al., 2016). The combination of ROCK inhibitors and PPAR agonists may simultaneously target metabolic disorders and vascular dysfunction.

Combination with glucagon-like peptide-1 (GLP-1) receptor agonists: GLP-1 receptor agonists (e.g., semaglutide) can reduce hepatic steatosis and inflammation, and even alleviate liver fibrosis in patients with MASH (Sanyal et al., 2025; Bansal et al., 2026). Combined with the effects of ROCK inhibitors on LSECs and HSCs, this combination strategy is expected to improve both metabolism and fibrosis.

Combination with targeted delivery systems: Combining ROCK inhibitors with vitamin A liposomes or M6P modification technology (Okimoto et al., 2019; van Beuge et al., 2011a; van Beuge et al., 2011b), together with other pathway inhibitors (e.g., TGF-β receptor inhibitors), can achieve multi-target, low-toxicity precision therapy.

In summary, anti-hepatic fibrosis intervention strategies targeting the Rho/ROCK signaling pathway encompass multiple approaches, including non-selective inhibitors, selective ROCK2 inhibitors, targeted drug delivery systems, natural products, exploration of new indications for marketed drugs, non-coding RNA regulation, and combination therapies (Table 2). Among these, the selective ROCK2 inhibitor TDI01 represents a promising candidate for clinical translation, with preliminary signals suggesting potential fibrosis improvement; however, rigorous Phase II/III trials with histological endpoints are needed to establish efficacy (Hu et al., 2026).

**TABLE 2:** Summary of anti-liver fibrosis drugs and intervention strategies targeting the Rho/ROCK signaling pathway.

Category	Drug/strategy	Target/mechanism	Representative models/research stage	Main effects	References
Non-selective ROCK inhibitors	Fasudil	ROCK1/2	Liver fibrosis mouse; diabetic rat models	Activates NK cells, inhibits HSCs activation and proliferation, reduces α-SMA/TGF-β1 expression; alleviates diabetes-associated liver fibrosis	( Han et al., 2021; Xie et al., 2018; Zhou et al., 2014 )
	Y-27632	ROCK1/2	Liver fibrosis rat; cirrhosis model; ischemia-reperfusion model	Inhibits HSCs activation markers (Na^+^/Ca^2+^ exchanger), reduces hydroxyproline content, improves hepatic sinusoidal microcirculation, enhances liver transplant survival rate	(Murata et al., 2003; Murata et al., 2001; Mizunuma et al., 2002; Mizunuma et al., 2003; Kuroda et al., 2012)
Selective ROCK2 inhibitors	KD025	ROCK2	Liver fibrosis mouse	Suppresses macrophage TNFα production, disrupts macrophage niches, interferes with STAT3/Cofilin signaling, reduces IL-17 levels	( Nalkurthi et al., 2022) (Nalkurthi et al., 2022 )
	GV101	ROCK2	Liver fibrosis mouse	Targets Akt-mTOR-S6K axis, downregulates pCofilin/pSTAT3/pAkt, upregulates pSTAT5/pAMPK, reduces collagen levels	(Zanin-Zhorov et al., 2023)
	TDI01	ROCK2	MASH rodent/mini-pig models; Phase I trial (ChiCTR2200058868); Extension trial (ChiCTR2400082056)	Restores LSECs phenotype, blocks angiocrine signals; Phase I confirms favorable safety; an extension trial shows a fibrosis regression trend in 5 of 6 patients	(Hu et al., 2026; Sun et al., 2026)
Targeted drug delivery systems	VA-liposome-ROCK inhibitor	HSCs vitamin A receptor	Liver fibrosis mouse	Achieves equivalent anti-fibrotic efficacy at ∼1/100 dose, avoids renal dysfunction	(Okimoto et al., 2019)
	VA-liposome-Y-27632	HSCs vitamin A receptor	Steatotic liver ischemia-reperfusion rat	Improves survival rate and hepatic blood flow, no severe hypotension	(Kuroda et al., 2015)
	M6PHSA-Y-27632	HSCs M6P/IGFII receptor	Liver fibrosis mouse	Enables HSCs-specific uptake, inhibits migration and contraction, significantly reduces collagen deposition	( van Beuge et al., 2011a; van Beuge et al., 2011b )
​	pPBHSA-Y-27632	HSCs PDGFβ receptor	Cirrhotic rat	Reduces portal pressure and intrahepatic resistance without affecting systemic blood pressure; provides additional renoprotective effects	(Klein et al., 2019)
Natural products	Ursolic acid	NOX4/ROS + RhoA/ROCK1	Liver fibrosis mouse; HSCs-T6 cells	Simultaneously inhibits both pathways, reverses liver fibrosis, improves gut microbiota dysbiosis	(Wan et al., 2020)
	Epigallocatechin-3-gallate	Rho signaling	TWNT-4 human HSCs line	Inhibits stress fiber formation and α-SMA expression, induces HSCs apoptosis	(Higashi et al., 2005)
	Artesunate	Promoting the ubiquitination and degradation of ROCK1 protein	LX2 human HSCs line; liver fibrosis mouse	Induces ferroptosis in HSCs, ameliorates hepatic fibrosis	(Wang et al., 2024)
	Obtusifolin	Inhibiting the ROCK2/Cofilin-1/NF-κB pathway	Liver fibrosis mouse	Attenuates hepatic inflammation and fibrosis	(Panneerselvam et al., 2026)
Approved drugs (repurposing)	Simvastatin	Inhibits RhoA prenylation via mevalonate pathway; RhoA/ROCK + Ras/ERK	MASH mouse	Attenuates hepatic inflammation and fibrosis	(Schierwagen et al., 2016)
	Atorvastatin	RhoA/ROCK inhibition + eNOS/PKG activation	Cirrhotic rat	Lowers portal pressure without affecting mean arterial pressure	(Trebicka et al., 2007)
	Carvedilol	AT1R/RhoA/ROCK2 pathway	Ang II-stimulated HSCs; liver fibrosis models	Inhibits HSCs proliferation and contraction, alleviates liver fibrosis	(Wu et al., 2019)
	Spironolactone	ROCK-2 inhibition + NO/PKG activation	BDL cirrhotic rat	Reduces portal hypertension	(Wu et al., 2019)
Non-coding RNAs	miR-125b antagomir	Stard13/RhoA (pro-fibrotic)	Liver fibrosis mouse	Antagonizing miR-125b significantly attenuates liver fibrosis	(You et al., 2018)
	miR-372-3p overexpression	RhoC (anti-fibrotic)	TGF-β1-activated LX-2 cells	Inhibits proliferation, promotes apoptosis, reduces COL I/α-SMA levels	(Ou et al., 2025)
	lncRNA NORAD knockdown	miR-511-3p/ROCK2 axis	MASLD mouse	Liver-specific NORAD knockdown significantly slows disease progression	(Zhang et al., 2025)
Combination therapy	ROCKi + FXR agonist	Obeticholic acid (OCA)	Preclinical/clinical	ROCK reduces portal pressure, and FXR exerts anti-inflammatory and anti-fibrotic effects	( Zobair et al., 2019; Eslam et al., 2019 )
​	ROCKi + PPAR agonist	Elafibranor	NASH clinical trial	Targets both metabolic disorders (PPAR) and vascular dysfunction (ROCK)	(Ratziu et al., 2016)
	ROCKi + GLP-1 receptor agonist	Semaglutide	NASH Phase III trial	Reduces steatosis/inflammation (GLP-1) + targets LSECs/HSCs (ROCK)	( Sanyal et al., 2025; Bansal et al., 2026 )

HSCs, hepatic stellate cell; LSECs, liver sinusoidal endothelial cell; MASH, metabolic dysfunction-associated steatohepatitis; NK, natural killer; α-SMA, alpha-smooth muscle actin; TGF-β, transforming growth factor-beta; TNFα, tumor necrosis factor α; STAT3, signal transducer and activator of transcription 3; NF-κB, nuclear factor kappa-B; eNOS, endothelial nitric oxide synthase; PKG, protein kinase G; NOX4, NADPH, oxidase 4; ROS, reactive oxygen species; VA, vitamin A; M6P, mannose-6-phosphate; IGFII, insulin-like growth factor II; PDGF, platelet-derived growth factor; OCA, obeticholic acid; GLP-1, glucagon-like peptide-1. Drugs in bold indicate the most advanced candidates in clinical development.

## 7 Research limitations, controversies, and future directions

Although the role of the Rho/ROCK pathway in liver fibrosis has been widely recognized, there are still several unresolved issues and controversies in this field.

### 7.1 Limitations of current pharmacological ROCK inhibitors

Before discussing isoform-specific controversies, it is important to acknowledge the pharmacological limitations of the tools most commonly used to study ROCK function in liver fibrosis. Y-27632 is widely used as a ROCK inhibitor in preclinical studies at concentrations of 10–30 μM. Its inhibitory constants (Ki) for ROCK1 and ROCK2 are 140–220 nM and 300 nM, respectively. However, Y-27632 also inhibits other AGC-family kinases at concentrations commonly used in cell-based assays: PRK2 (IC_50_ ≈ 600 nM; only ∼3–4-fold selectivity over ROCK1), PKN (Ki ≈ 3.1 μM), citron kinase (Ki ≈ 5.3 μM), PKA (Ki ≈ 25 μM), and PKCα (Ki ≈ 73 μM) (Davies et al., 2000; Ishizaki et al., 2000). The selectivity over MLCK (>250 μM) and PAK (unaffected at ∼100 μM) is more favorable. These off-target activities, particularly the relatively potent PRK2 inhibition, mean that many 'ROCK-dependent' phenotypes observed in Y-27632 studies may partially reflect inhibition of other AGC kinases.

Fasudil (HA-1077), approved in Japan for cerebral vasospasm, was originally developed as a vasodilator. In addition to ROCK, fasudil inhibits PKA (Ki ≈ 3.6 μM), PKC, and MLCK at concentrations relevant to its clinical use (Breitenlechner et al., 2003). The clinical implications include systemic hypotension, which limits the achievable dose in liver fibrosis patients and complicates the interpretation of antifibrotic effects observed in preclinical models.

Most current ROCK inhibitors distribute broadly across tissues following systemic administration, which limits their utility for cell-specific targeting. This lack of selectivity contributes to systemic side effects and makes it difficult to attribute observed antifibrotic effects to actions in specific cell types. This limitation provides additional rationale for the cell-targeted delivery strategies discussed in Section 6. Recent advances in targeted nanoparticle delivery, such as salvianolic acid B-loaded albumin nanoparticles that inhibit the HSC RhoA/ROCK pathway while reducing portal hypertension without systemic vasodilation (Wang et al., 2026), exemplify strategies to overcome this limitation.

Systemic ROCK inhibition affects vascular tone and blood pressure regulation through actions on vascular smooth muscle cells and endothelial cells (Hu et al., 2026; Wang et al., 2025). In the context of liver fibrosis treatment, this is particularly relevant because cirrhotic patients often have portal hypertension, where systemic vasodilation may have complex hemodynamic consequences, and the liver receives dual blood supply (hepatic artery and portal vein), making the hemodynamic effects of systemic ROCK inhibition difficult to predict (Zeng et al., 2026).

ROCK inhibition also affects multiple aspects of immune cell function, including T cell activation (Th17/Treg balance), NK cell cytotoxicity, and macrophage polarization. These effects may have both beneficial (anti-inflammatory) and potentially detrimental (immunosuppressive) consequences in the context of chronic liver disease.

### 7.2 Controversy 1: dual role of ROCK signaling in hepatocytes

Available evidence shows that activation of hepatocyte ROCK on the one hand promotes lipid synthesis and steatosis (mediated by ROCK1), and on the other hand protects hepatocyte survival during acute liver injury (co-mediated by ROCK1/2) (Dohnalkova et al., 2023; Herrera et al., 2025). This seemingly contradictory function suggests that systemic ROCK inhibition may aggravate hepatocyte injury while improving fibrosis, especially in patients with preexisting liver insufficiency. Future studies need to develop hepatocyte-specific ROCK knockout or knock-in models to precisely distinguish the functional switching mechanisms under different stimuli. In addition, the development of cell type-specific targeted ROCK inhibitors (e.g., via LSECs- or HSCs-specific ligand conjugation) may be a strategy to avoid the loss of hepatocyte protective functions.

As analyzed in Section 4.2, three key factors may reconcile these observations: the temporal dimension (acute vs. chronic activation), the activity threshold (biphasic dose-response), and the distinction between combined ROCK1/ROCK2 deletion and selective ROCK1 inhibition (Dohnalkova et al., 2023; Tak et al., 2022; Herrera et al., 2025). An additional consideration specific to clinical translation is that patients with MASH-related fibrosis often have underlying metabolic dysfunction that may alter the risk–benefit calculus of ROCK inhibition—specifically, the hepatoprotective functions observed in acute injury models may be less relevant in the chronic metabolic context where ROCK1-mediated steatosis predominates. Future clinical protocols should therefore stratify patients by etiology and baseline liver function to identify subpopulations most likely to benefit from selective ROCK2 inhibition while retaining hepatocyte ROCK1 activity.

### 7.3 Controversy 2: bidirectional regulation of HSCs migration by ROCK inhibitors

Low-concentration ROCK inhibitors inhibit HSCs migration, whereas high concentrations promote migration instead (Yanase et al., 2003; Bachtler et al., 2023). This phenomenon may be related to differences in cytoskeletal reorganization patterns at different concentrations, but its molecular mechanism remains unclear. Since drug concentrations may be heterogeneous in the fibrotic liver, this bidirectional regulation may affect actual therapeutic efficacy. Future studies need to systematically investigate the dose-effect relationships of different ROCK inhibitors (fasudil, Y-27632, KD025, TDI01) on HSCs migration, contraction, and collagen production at different concentrations to optimize dosing regimens.

### 7.4 Main barriers to clinical translation

Although the Rho/ROCK pathway demonstrates significant potential in the treatment of liver fibrosis, its clinical translation faces multiple challenges, including hypotension, loss of hepatocyte protective function, unknown long-term safety profiles, and etiological heterogeneity (Table 3).

**TABLE 3:** Major obstacles and potential solutions for clinical translation.

Barrier	Description	Potential solutions
Hypotension	Non-selective ROCK inhibitors can reduce arterial pressure	Selective ROCK2 inhibitors (KD025, TDI01) have lower hypotension risk; targeted delivery systems
Loss of hepatocyte protection	Systemic inhibition may impair hepatocyte tolerance to injury	Cell-specific targeting (LSECs or HSCs targeting)
Unknown long-term safety	Short follow-up duration in existing clinical trials	Extend follow-up periods and monitor liver function, renal function, and cardiovascular events
Etiological heterogeneity	ROCK function may differ across etiologies (MASH, viral, alcoholic)	Conduct subgroup analyses for different etiologies
Preliminary clinical evidence	TDI01 clinical data limited to small uncontrolled study (n = 6)	Rigorous Phase II/III RCTs with histological endpoints needed

### 7.5 Future research directions

1. In-depth dissection of isoform-specific mechanisms: Use conditional gene knockout mice to clarify the precise functions of ROCK1 and ROCK2 in HSCs, LSECs, hepatocytes, and macrophages.
2. Cell type-specific targeted strategies: Develop HSCs/LSECs-specific drug delivery systems based on single-chain antibodies or ligands to precisely deliver ROCK inhibitors to pathogenic cells.
3. Combination therapy clinical trials: Design combination regimens of ROCK inhibitors with FXR agonists, PPAR agonists, or GLP-1 receptor agonists, and evaluate synergistic effects and safety.
4. Single-cell omics to reveal heterogeneity: Use single-cell RNA sequencing and spatial transcriptomics to map the dynamic changes of the ROCK signaling pathway in different cell subsets during fibrosis progression.
5. Development of proteolysis-targeting chimera (PROTAC) degraders: Compared with kinase inhibitors, PROTAC technology can achieve complete degradation of the ROCK protein, which may produce more durable efficacy while reducing off-target effects.
6. Non-coding RNA therapies: Novel nucleic acid drugs based on miR-125b antagonists or lncRNA NORAD inhibitors can specifically regulate the Rho/ROCK pathway and have therapeutic potential.

## 8 Unanswered questions

Are the functions of ROCK1 and ROCK2 interchangeable at different stages of HSCs activation? Current evidence mainly comes from static inhibition experiments, and studies on time-specific knockout are lacking.

Will inhibition of hepatocyte protective function by ROCK inhibitors lead to impaired liver regeneration in chronic liver disease? Long-term animal experiments are needed for verification.

Are there differences in the activation degree and isoform dependence of the ROCK pathway in liver fibrosis of different etiologies (MASH vs. viral hepatitis vs. alcoholic)? Head-to-head comparisons are currently lacking.

Will targeted delivery of ROCK inhibitors to HSCs affect the normal physiological functions of ROCK2 in LSECs (e.g., vascular tone regulation)? More refined cell-specific pharmacokinetic studies are needed.

Will the combination of ROCK inhibitors and immune checkpoint inhibitors (e.g., anti-PD-1) increase hepatotoxicity? Given the role of ROCK in T cell function, this issue deserves attention.

As discussed in Section 2.2, transcriptomic approaches including scRNA-seq can delineate isoform-specific expression patterns but cannot distinguish catalytically active from inactive ROCK pools. Confirming pathway activation in specific cell types requires complementary phosphoproteomic or functional readout studies (e.g., p-MYPT1, p-MLC, *in situ* kinase assays). The development of isoform-specific FRET biosensors suitable for fixed or clinical tissue samples would be a valuable addition to the current toolkit.

## 9 Conclusion

The Rho/ROCK signaling pathway plays a multi-level, multi-cell-type core regulatory role in the occurrence and development of liver fibrosis. From the activation, contraction, migration, and proliferation of HSCs, to the regulation of angiocrine function of LSECs, to hepatocyte lipid metabolism and survival, as well as inflammatory response regulation of macrophages and immune cells, the Rho/ROCK pathway participates in almost every key link of liver fibrosis through the differential functions of its two isoforms (ROCK1 and ROCK2). The interactive regulatory network of this pathway with multiple signaling pathways, including TGF-β/Smad, Wnt/β-catenin, Hippo/YAP, NOX4/ROS, PI3K/Akt, MAPK, and cAMP/PKA, further enriches the molecular regulatory mechanism of liver fibrosis.

Therapeutic strategies targeting the Rho/ROCK pathway have made remarkable progress. From non-selective inhibitors such as fasudil and Y-27632, to selective ROCK2 inhibitors such as KD025, GV101, and TDI01, and then to targeted drug delivery systems such as vitamin A-coupled liposomes, M6PHSA, and pPBHSA, this field presents a multi-level and multi-directional development trend. Among these, the selective ROCK2 inhibitor TDI01 has entered clinical trials and shown preliminary signals of fibrosis improvement; however, rigorous randomized controlled trials with larger sample sizes and predefined histological endpoints are needed to establish clinical antifibrotic efficacy.

However, the dual role of ROCK signaling in hepatocytes (promoting lipid metabolism vs. protecting survival) suggests that systemic ROCK inhibition may bring unexpected risks, and the development of cell-specific targeted strategies and combination regimens is the future direction. With an in-depth understanding of isoform-specific functions and signaling interaction networks, as well as the advancement of novel drug delivery technologies and clinical trials, therapeutic strategies targeting the Rho/ROCK pathway have the potential to establish a new paradigm for precision antifibrotic therapy, moving beyond symptom management toward mechanism-based disease modification.

## Glossary

α-SMA: α-smooth muscle actin
Akt: protein kinase B
Ang II: angiotensin II
AT1R: angiotensin type 1 receptor
BDL: bile duct ligation
cAMP: cyclic adenosine monophosphate
**CCl**_**4**_: Carbon tetrachloride
COL1A1: collagen type I alpha one chain
CTGF: connective tissue growth factor
ECAD: E-cadherin
ECM: extracellular matrix
eNOS: endothelial nitric oxide synthase
ERK: extracellular signal-regulated kinase
ET-1: endothelin-1
FXR: farnesoid X receptor
GLP-1: glucagon-like peptide-1
Gli1: glioma-associated oncogene one
HSCs: hepatic stellate cells
IGFII: insulin-like growth factor II
IL-17: interleukin-17
LIMK: LIM kinase
lncRNA: long non-coding RNA
LSECs: liver sinusoidal endothelial cells
M6P: mannose-6-phosphate
M6PHSA: mannose-6-phosphate-modified human serum albumin
MAFLD: metabolic dysfunction-associated fatty liver disease
MAPK: mitogen-activated protein kinase
MASH: metabolic dysfunction-associated steatohepatitis
miRNA: microRNA
MLC: myosin light chain
MLCK: myosin light chain kinase
MLCP: myosin light chain phosphatase
MMP: matrix metallopeptidase
mTOR: mechanistic target of rapamycin kinase
MYPT1: myosin-binding subunit of myosin light chain phosphatase
NF-κB: nuclear factor-κB
NK: natural killer
NOX4: NADPH oxidase 4
OCA: obeticholic acid
PDGF-BB: platelet-derived growth factor-BB
PI3K: phosphoinositide 3-kinase
PKA: protein kinase A
PPAR: peroxisome proliferator-activated receptor
pPBHSA: PDGFβ receptor-recognizing peptide-modified human serum albumin
PROTAC: proteolysis-targeting chimera
Rac1: Ras-related C3 botulinum toxin substrate one
RIP3: receptor-interacting protein 3
ROCK: Rho-associated coiled-coil containing protein kinase
ROS: reactive oxygen species
S1P: sphingosine-1-phosphate
S1PR2: sphingosine-1-phosphate receptor 2
S6K: ribosomal protein S6 kinase
Smad: suppressor of mother against decapentaplegic
STAT3: signal transducer and activator of transcription 3
Sufu: suppressor of fused homolog
TAA: thioacetamide
TCF4: transcription factor 4
TGF-β: transforming growth factor-β
TLR4: Toll-like receptor 4
TNF-α: tumor necrosis factor α
Wnt: Wingless-Int1
YAP: Yes-associated protein
